# ARF: a versatile DNA damage response ally at the crossroads of development and tumorigenesis

**DOI:** 10.3389/fgene.2014.00236

**Published:** 2014-07-22

**Authors:** Athanassios Kotsinas, Panagiota Papanagnou, Konstantinos Evangelou, George C. Trigas, Vassiliki Kostourou, Paul Townsend, Vassilis G. Gorgoulis

**Affiliations:** ^1^Molecular Carcinogenesis Group, Department of Histology and Embryology, School of Medicine, University of AthensAthens, Greece; ^2^Vascular Adhesion Lab, Biomedical Sciences Research Center Alexander FlemingAthens, Greece; ^3^Faculty Institute of Cancer Sciences, University of Manchester, Manchester Academic Health Science CentreManchester, UK; ^4^Manchester Centre for Cellular Metabolism, University of Manchester, Manchester Academic Health Science CentreManchester, UK; ^5^Biomedical Research Foundation, Academy of AthensAthens, Greece

**Keywords:** ARF, ATM, angiogenesis, ocular development, vascular network, involution, meiosis, spermatogenesis

## Abstract

Alternative reading frame (ARF) is a tumor suppressor protein that senses oncogenic and other stressogenic signals. It can trigger p53-dependent and -independent responses with cell cycle arrest and apoptosis induction being the most prominent ones. Other ARF activities, particularly p53-independent ones, that could help in understanding cancer development and provide potential therapeutic exploitation are underrated. Although ARF is generally not expressed in normal tissues, it is essential for ocular and male germ cells development. The underlying mechanism(s) in these processes, while not clearly defined, point toward a functional link between ARF, DNA damage and angiogenesis. Based on a recent study from our group demonstrating a functional interplay between ataxia-telangiectasia mutated (ATM) and ARF during carcinogenesis, we discuss the role of ARF at the crossroads of cancer and developmental processes.

## INTRODUCTION

The ARF (p14^ARF^ in humans, p19^ARF^ in mice) tumor suppressor is encoded by the *INK4A/ARF* locus that also harbors another onco-suppressor, namely the cyclin-dependent kinase inhibitor p16^INK4A^ (Quelle et al., 1995; Sherr, 2006). The p16^INK4A^ protein maintains pRB in an active form to inhibit E2F activity (Tsantoulis and Gorgoulis, 2005; Sherr, 2006). In this way S-phase entry and therefore cell division is prevented (Sherr, 2006). On the other hand, ARF is a “sensor” of various stresses including oncogenic ones, like aberrant expression of Myc, E1A and RAS (de Stanchina et al., 1998; Zindy et al., 1998; Palmero et al., 1999). Other stresses that can also activate ARF are oxidative stress and heat shock (Damalas et al., 2011; Liontos et al., 2012). In response it can act both in p53-dependent and -independent manners (Weber et al., 2000; Kotsinas et al., 2014), triggering either growth arrest or apoptosis to counteract abnormal cell proliferation (Sherr, 2006). Apart from cancer (Sherr, 2006), accumulating data highlight ARF as a versatile protein implicated in various physiological processes including developmental ones (Thornton et al., 2005; Gromley et al., 2009; Churchman et al., 2011), immunomodulation (Través et al., 2012) and ribosomal ribonucleic acid (rRNA) maturation (Sugimoto et al., 2003), as well as pathological ones, such as atherogenesis (González-Navarro et al., 2010). Most of the best known ARF functions are p53-dependent ones (Sherr, 2006), while independent activities seem to be underrated.

Deficiency of ARF or p53, has revealed different phenotypes in mice. Specifically, ARF-null animals mainly develop sarcomas, whereas p53-null animals are predominantly characterized by the evolvement of lymphomas (Kamijo et al., 1999). This finding was among the first experimental indications that ARF and p53 can signal independently of each other and not necessarily in a strict linear signaling pathway. Therefore, they may fulfill different tasks in tumor surveillance. Moreover, recent evidence from our group has highlighted the functional significance of a cross-talk among ARF and ATM (Velimezi et al., 2013; Kotsinas et al., 2014) and how ARF can act as an “auxiliary” tumor suppressive mechanism throughout cancer progression in case the DDR pathway is compromised (Velimezi et al., 2013).

In various normal tissues ARF is not expressed. Striking exceptions are the developing oculus (eye), testicular tissue and umbilical arteries (Thornton et al., 2005; Freeman-Anderson et al., 2009; Gromley et al., 2009; Churchman et al., 2011). The underlying mechanism(s) taking place in these tissues, while not clearly defined, point toward a functional link between ARF, DNA damage and angiogenesis.

Considering the ATM and ARF interplay in carcinogenesis (Velimezi et al., 2013), we discuss in this article the role of ARF at the crossroads of cancer and developmental processes. We present the current knowledge regarding the role of ARF in development, particularly during spermatogenesis and ocular development in mice. Furthermore, we provide data (including unpublished ones) consolidating the notion that the interference with vascular dynamics accounts for a novel, inherent p53-independent tumor suppressive property of ARF that could be therapeutically exploited in p53-deficient tumors.

## THE ROLE OF ARF IN MALE GERM CELL DEVELOPMENT: A MATTER OF PRESERVING GENOMIC INTEGRITY

**FIGURE 1:**
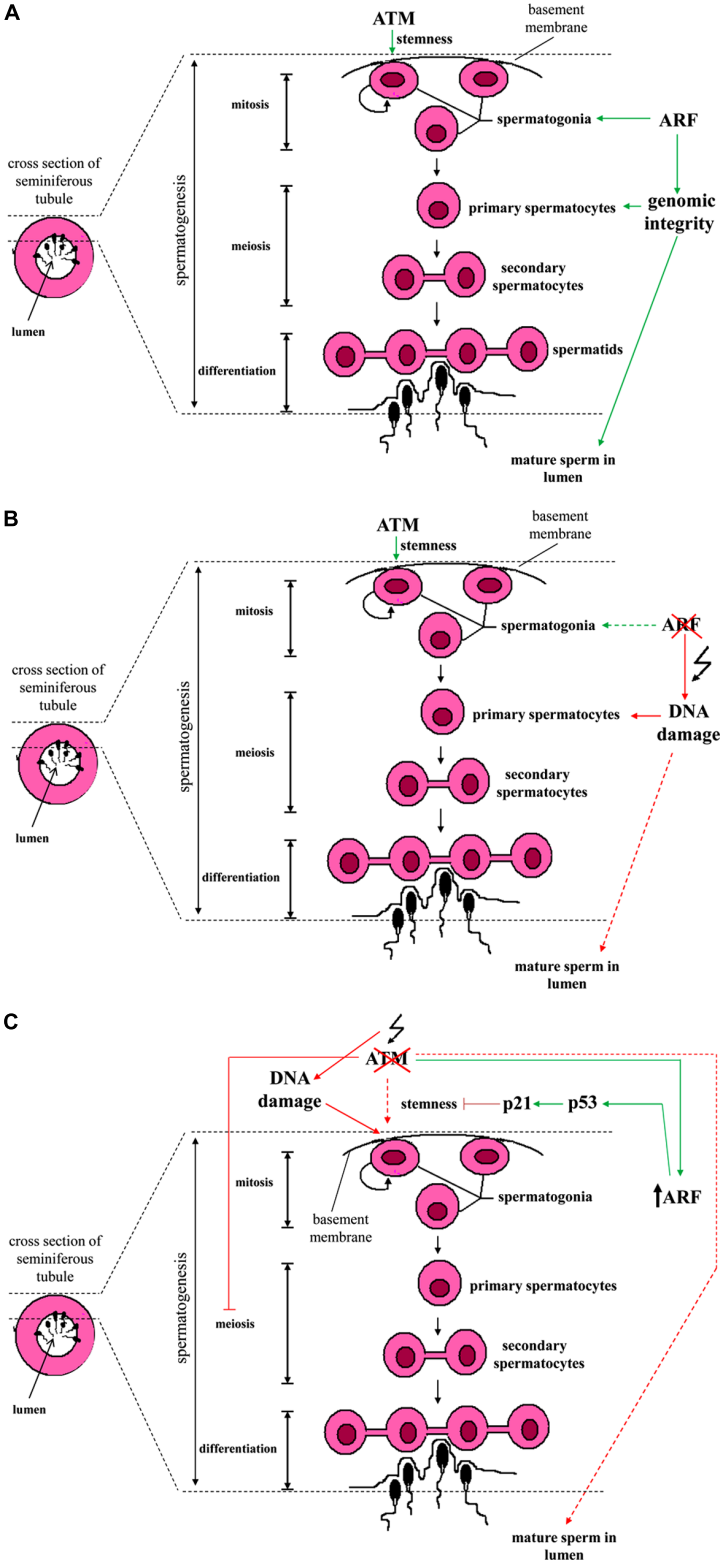
**Schematic presentation of the spermatogenesis stages across the wall of a seminiferous tubule and the interrelations with ATM and ARF. (A)** Spermatogonia are found in close proximity to the basement membrane of a seminiferous tubule. A subpopulation of spermatogonia exhibits stem cell ability and self-renews via mitotic divisions. ATM kinase is essential for their stemness (symbolized by a semicircular shape). Some of the spermatogonia eventually differentiate into primary spermatocytes. The latter, undergo meiosis to give rise to secondary spermatocytes which in turn, form spermatids (connected via cytoplasmic bridges). Spermatids engage a series of cytodifferentiative programs and sperm is finally formed. ARF expression in spermatogonia is required in order to prevent the occurrence of DNA damage in meiotic primary spermatocytes (at the stage of pachytene) through participating in a feed-forward program. **(B)** In the absence of ARF, genomic integrity in primary spermatocytes is threatened and the production of mature sperm released in the lumen is compromised. **(C)** Upon ATM deficiency, spermatogonia loose their genomic integrity and ARF undergoes upregulation. Consequently, an ARF-mediated p53/p21^WAF1/Cip1^ growth restrictive pathway counteracts spermatogonial stemness. Due to the fact that ATM deficiency hampers normal spermatocytic meiotic progression (at prophase I), sperm production is compromised (Barlow et al., 1996, 1998; Xu et al., 1996). The different subtypes of spermatogonial cells, Sertoli cells that support spermatogonia, stages and phases of meiosis, the different stages of spermatid differentiation as well as ploidy of cells are not shown here for reasons of simplicity (Colored lines depict ATM and/or ARF effects on spermatogenesis. Dashed colored lines denote weak effect or weak activation. Red lines represent adverse effect, while green ones correspond to physiologic functions).

Spermatogenesis is a spatio-temporally coordinated process by which undifferentiated spermatogonia (i.e., the stem cell population of germinal cells residing on the basement membrane of semiferous tubules) evolve into spermatocytes in the lumen through a series of mitotic and meiotic cellular divisions (Cooke and Saunders, 2002). In mice, Gromley et al. (2009) reported that in this developmental process ARF is selectively expressed in mitotic spermatogonia, but not in intratubular spermatocytes that stain positive for meiotic markers (**Figure 1**). Intriguingly, in the absence of ARF the testicles of mice exhibit atrophy and produce much lower quantity of sperm compared to wild-type animals. These phenotypic changes are accompanied by increased levels of apoptosis in germ cells during all their developmental stages. Of note, abolishment of this transient expression of ARF during spermatogenesis is sufficient to compromise this developmental process throughout the whole reproductive life of mice (Gromley et al., 2009).

Subsequent experiments carried out by the same research group demonstrated that ARF is essential for normal meiotic progression and survival of spermatocytes via initiation of a feed-forward program in their progenitors, the spermatogonia. Interestingly, ARF expression in spermatogonia did not exert an anti-proliferative effect, as they also expressed cyclin D1. Testicular atrophy and reduced production of mature sperm in ARF-deficient mice was not mechanistically related to a disturbed pituitary-gonadal axis and deregulated levels of circulating FSH or LH. Rather, in the absence of ARF a marked increase in the number of spermatocytes undergoing p53-dependent apoptosis at the stage of pachytene of prophase I was observed (Churchman et al., 2011).

Of note, during HR in meiosis the topoisomerase-II like Spo11 enzyme normally causes DSBs, which in turn trigger the activation of ATM and the generation of γ-H2AX foci selectively at the leptotene and zygotene stages (Inagaki et al., 2010). At the pachytene stage when synapsis of homologous chromosomes has been completed, γ-H2AX foci are normally not detected. However, *in situ* analyses showed that in an ARF deficient background, the number of γ-H2AX foci in pachytene spermatocytes is significantly increased (Churchman et al., 2011). The latter observation was therefore suggestive of meiotic defects that have deleterious effects on spermatocytic genomic integrity. Additional evidence for meiotic abnormalities included the identification of asynaptic regions as well as decreased number of foci of the Rad51 and Dmc1 recombinases known to be associated with the repair of DSBs that occur during HR. Overall, the authors concluded that ARF through a yet poorly understood mechanism, interferes with HR to preserve the fidelity of meiosis in spermatocytes and to protect them from DNA damage and p53-dependent apoptosis (Churchman et al., 2011). Whether the apoptotic death of spermatocytes is a consequence of DNA damage *per se* or not, remains to be elucidated. Inhibition rather than induction of p53-dependent apoptosis by ARF is a unique feature of male germ cells and reveals an opposite to the well-established p53-mediated pro-apoptotic role of ARF in somatic cells (Lowe and Sherr, 2003).

Importantly, ARF is not the only DNA damage-related protein that interferes with the spermatogonial program (**Figure 1**). The DDR kinase ATM is actually essential for the maintenance of undifferentiated spermatogonia and for retaining their stemness (Barlow et al., 1998; Takubo et al., 2008). In murine testicles, ATM deficiency progressively results in the depletion of undifferentiated spermatogonia. This is functionally associated with cell cycle arrest, loss of genomic integrity and defects at the pre-meiotic level (Barlow et al., 1996, 1998; Elson et al., 1996; Xu et al., 1996; Takubo et al., 2008). Mechanistically, the absence of ATM is linked to the accumulation of DNA damage and the activation of an ARF/p53/p21^WAF1/Cip1^ – dependent growth restrictive pathway. Notably, in transplantation assays where spermatogonia are delivered into the seminiferous tubules of mutant mouse strains that exhibit defective spermatogenesis, p21^WAF1/Cip1^ deficiency is able to restore spermatogonial repopulation ability in an ATM-null background (Takubo et al., 2008).

Taken together, all the above data pinpoint that both ARF and ATM are critical factors for the maintenance of spermatogonia and survival of their progeny during male germ cell development (**Figure 1**; Barlow et al., 1996; Takubo et al., 2008; Churchman et al., 2011). This common feature parallels with their same function as tumor suppressors in somatic cells. Nevertheless, the imposed outcomes are different in each cell type, suggesting a functional bimodality. In somatic cells ATM and ARF induce cell growth restrictive or apoptotic routes, whereas in spermatogonia they do not interfere with their ability to proliferate. It is rather their depletion that leads to such cellular responses. A further issue, stemmed from the ability of ATM to regulate ARF turnover (Velimezi et al., 2013), is whether this functional link may operate in male germ cells. As demonstrated, in response to irradiation ARF protein in spermatogonia is markedly down-regulated (Velimezi et al., 2013). Evidence was also provided by Takubo et al. (2008) showing that in ATM null spermatogonia there is a higher activation of ARF, supporting the interconnection between ATM and ARF. Nevertheless, details on how this link endorses this developmental process require further clarifications.

## ARF AS A REGULATOR OF THE VASCULAR NETWORK IN DEVELOPMENT AND TUMORIGENESIS

Apart from the male germ cell development in mice as presented above, ARF also plays a central role in the murine ocular development. In mice models it was shown that ARF is required for the maturation of the primary vitreous into the secondary vitreous; an avascular jelly like substance within the developing oculus. The expression of ARF in the vitreous is postnatally induced up to P5, in order to trigger the involution of HVS, a transient anatomical entity in the developing oculus that has to regress during P6–P10 (McKeller et al., 2002). ARF promotes HVS involution through restricting the accumulation of mural cells that cover the vessels and contributes to the preservation of their stability in a PDGFRβ-dependent/p53-independent manner (Silva et al., 2005; Gromley et al., 2009). Overall, two models have been proposed to explain the mechanistic basis by which ARF controls the vascular network dynamics. According to the first model, when ARF is induced by unknown yet upstream developmental signals it suppresses PDGFRβ expression via uncharacterized mediators. In this way, ARF restricts mural cell proliferation. In the second scenario, ARF acts as a cell fate determinant during the maturation of mural cells to shut off PDGFRβ expression and force them to differentiate into a type of perivascular cells that selectively support transient vessels (Thornton et al., 2005).

Notably, phenotypic characteristics exhibited by ARF deficient mice are also found in the developmental human ocular disease termed PHPV and include microphthalmia and degenerative alterations in lens (cataractogenesis; McKeller et al., 2002). Overall, these data pinpoint to the existence of an ARF-mediated tightly regulated spatio-temporal angiogenic developmental process. It appears that ARF’s role in vascular evolvement is not solely restricted to developmental processes. Rather, data corroborate the notion that ARF exhibits a wider activity in the control of vascular dynamics, which are functionally linked to tumor progression.

“Angiogenic switch” is a prominent feature of tumor progression (Bergers and Benjamin, 2003). In line with the notion that ARF plays a wide role in the modulation of pathways affecting vasculature, an inverse correlation among MVD and ARF in human clinical colon cancer samples has been reported (Kawagishi et al., 2010). Furthermore, ARF/p53 deficient MEFs challenged with oncogenic RAS^V12^ when injected as xenografts produce tumors that grow faster relative to similarly treated cells, but retrovirally infected with ARF (Kawagishi et al., 2010). Tumor sections showed in the latter case a lower immunostaining for CD31, a neovascular marker. Exploring mechanistically their findings Kawagishi et al. (2010) determined that ARF suppresses the expression of VEGFA in a p53-independent manner in mouse cell lines. This involves the inhibition of VEGFA translation via the IRES of VEGFA.

Based on our recent finding that ATM controls ARF turnover (Velimezi et al., 2013), we sought to expand these observations in human tumors with inactive p53 and explore for potential therapeutic utilization. Treating the NSCLC cell line H1299 and cervical carcinoma HeLa cells, which do not express functional p53, with the ATM kinase inhibitor Ku55933 in order to stabilize ARF (Velimezi et al., 2013) we observed an inverse relationship among the expression of ARF and VEGF protein (**Figure S1A**). VEGF plays a central role in tumor angiogenesis (Crinò and Metro, 2014), while ARF can signal in a p53-independent fashion and hence, serve as a “back-up” barrier to tumorigenesis in case that p53 is inactivated (Velimezi et al., 2013). Therefore, upregulating ARF via inhibition of ATM activity may be exploited in p53-deficient human tumors as a novel anti-angiogenic therapeutic approach. In a next step, we investigated whether ARF opposes tumor angiogenesis *in vivo*. To address this issue H1299 cells were xenografted in immunocompromised mice. Sections from the generated tumors were stained with an antibody specific for the endothelial marker CD31, to evaluate MVD. As shown in **Figure S1B**, MVD is markedly decreased (>twofold) in tumors injected with a lentiviral vector expressing shRNA targeting ATM (ctl-shRNA/Lenti-shATM); a manipulation which upregulates ARF (Velimezi et al., 2013). The observed reduction in MVD was actually an ARF-dependent phenomenon, because MVD in H1299-shARF xenografts upon knocking down of ATM (shARF/Lenti-shATM) was found to be comparable to that estimated in ARF-expressing ones without lentivirus-mediated silencing of ATM kinase (ctl-shRNA/Lenti-ctl). Therefore, stabilization of ARF in the absence of ATM activity exhibits anti-angiogenetic effects *in vivo*, corroborating the claim that this p53-independent ATM/ARF axis could be therapeutically harnessed.

In our experiments we did not examine whether the observed ARF-mediated down-regulation of VEGF levels is associated with an inhibition of IRES-(in)dependent translation of VEGF transcript (Kawagishi et al., 2010), or if it is due to a translation-independent mechanism. For instance, HIF1α is a well-known transcriptional activator of VEGF and human ARF has been demonstrated to mediate the nucleolar sequestration of HIF1α, thereby hindering its ability to drive the expression of its target genes (Fatyol and Szalay, 2001). It would be a challenging future task to uncover the whole mechanistic spectrum that underlies the observed mutual exclusive expression among ARF and VEGF.

In a murine model of multi-stage pancreatic neuroendrocrine tumorigenesis where the SV-40 T-antigen is expressed in β cells, ARF deficiency was found to significantly accelerate tumor progression through promoting the angiogenic switch (Ulanet and Hanahan, 2010). In the absence of ARF the tumor burden was increased fivefold along with a higher number of angiogenic lesions. From a mechanistic perspective, in this malignancy ARF seems to act via engaging mainly p53-independent routes, while VEGF was not involved (Ulanet and Hanahan, 2010). A more recent study showed that ARF blocks the development of angiosarcomas associated with the exposure to the carcinogen urothene, possibly in a p53-dependent fashion (Busch et al., 2012). Intriguingly, it was hypothesized that ARF affects the proliferation of endothelial cells in adult mice. Hence, the inhibition of tumor angiogenesis and vascular malignancy possibly represent two discrete aspects of ARF’s tumor-suppressive activity.

## CONCLUSIONS AND PROSPECTS

**FIGURE 2:**
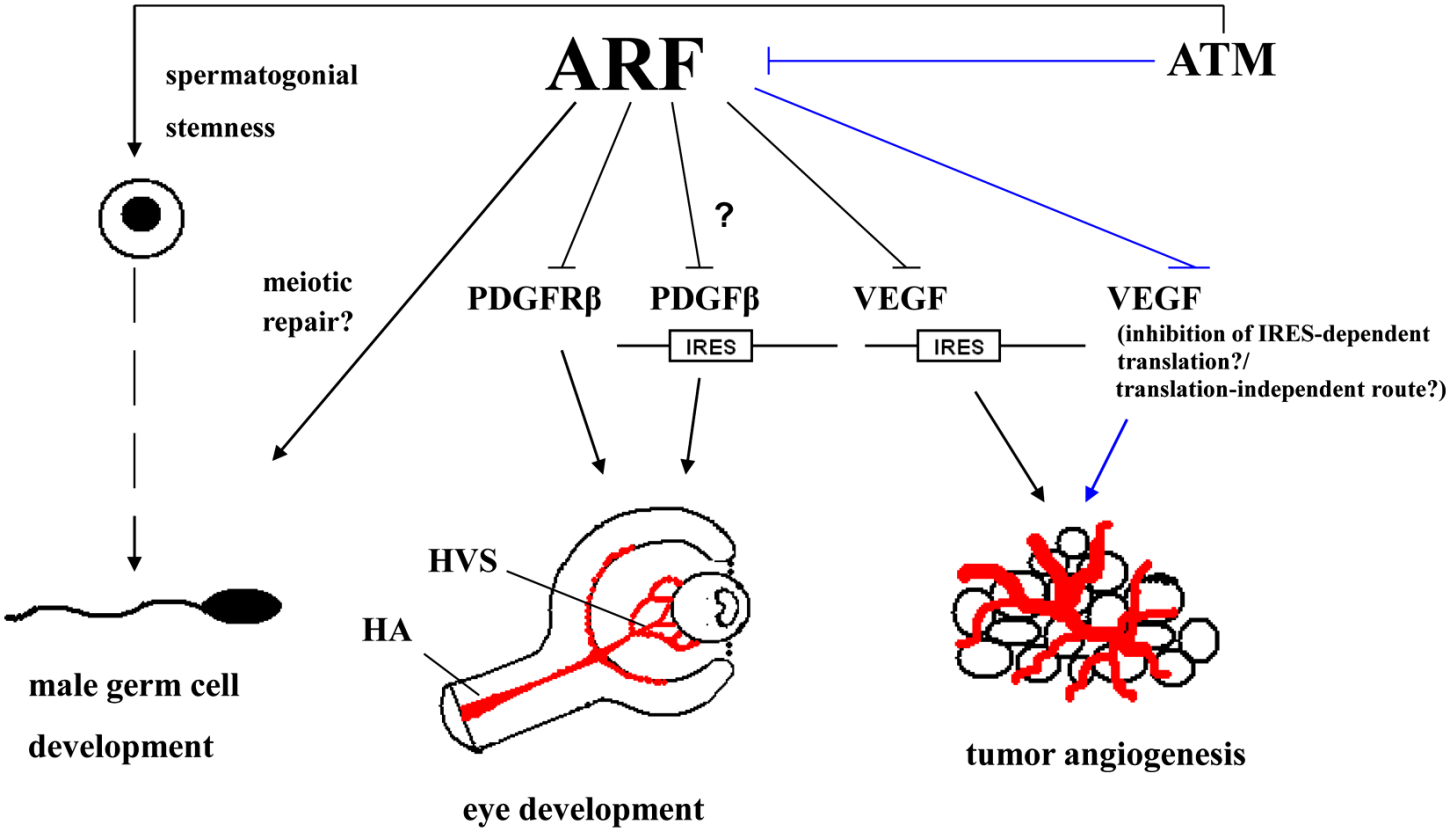
**ARF impinges both on developmental processes (male germ cell development and ocular development in mice) and tumor angiogenesis.** ARF is essential for normal spermatogenesis in mice possibly through interacting with the meiotic repair machinery. In the ocular development of the mouse, ARF is required for the involution of the hyaloid vascular system (HVS); a transient network of vessels that provides nutrients to the developing oculus. This is accomplished via a pathway in which ARF blocks platelet-derived growth factor receptor β (PDGFRβ)-dependent signaling which in turn, is necessary for the investment of vessels by mural cells and their maintenance. This may also be possibly regulated via the ARF-dependent inhibition of internal ribosome entry site (IRES)-mediated translation of PDGFRβ. ARF can also suppress the IRES-mediated translation of vascular endothelial growth factor A (VEGFA), thereby inhibiting tumor angiogenesis. Unpublished data indicate that the stabilization of endogenous ARF upon inhibition of ataxia-telangiectasia mutated (ATM) kinase activity can also result in a decrease in VEGF levels; although it is not known whether the underlying mechanism involves the suppression of IRES-mediated translation of VEGF or an IRES-independent route. It may even involve a route that is not associated with the control of VEGF at the translational level. (The ATM/ARF/VEGF pathway is shown by lines.) ATM kinase itself, independently of ARF, has also been incriminated in pathological angiogenesis in adult mouse oculus and cancer (Kerr and Byzova, 2012), but these effects are not depicted here for reasons of clarity. (HA, hyaloid artery.)

Collectively, we highlight underrated functions of ARF, positioned at the crossroads of tumor suppression (Weber et al., 2000; Tago et al., 2005; Kawagishi et al., 2010) and development (McKeller et al., 2002; Churchman et al., 2011; **Figure 2**), that could be exploited at the therapeutic level, especially in tumors with non-functional p53.

ARF as an onco-suppressor impedes carcinogenesis not only through interfering with cell proliferation and induction of apoptosis, but also via affecting other cancer-promoting processes such as angiogenesis (Kawagishi et al., 2010; Ulanet and Hanahan, 2010). In this context, in tumors with non-functional p53 the potential to upregulate ARF in an ATM manner (Velimezi et al., 2013), could be utilized as an anti-angiogenic “tool” in cancer management (**Figure S1**). This prospective therapeutic modality may be enhanced if combined with anti-VEGF or other anti-angiogenic factors, like tyrosine kinase inhibitors, that are currently used (Ferrara et al., 2004; Randall and Monk, 2010; Eisen et al., 2012). On one hand, such a dual treatment might lead to a synergistic outcome, possibly lethality, and on the other hand, it may allow the reduction of the administration doses of such compounds to avoid side-effects (Randall and Monk, 2010; Eisen et al., 2012). It should be noted that although the ATM inhibitor Ku55933 is highly selective toward ATM, its bioavailiability is low due to its pharmacokinetic properties (Golding et al., 2009). However, new ATM inhibitors, such as KU-60019, exhibiting a higher pharmacological profile have been released (Golding et al., 2009), rendering the proposed therapeutic approach feasible.

Another option in order to therapeutically exploit the aforementioned ARF/VEGF pathway would be the usage of synthetic ARF peptides comprising ARF’s amino-terminal residues 2–14 that mediate all the biological effects of ARF, including the anti-growth ones (Saporita et al., 2007). The therapeutic exploitation of the p53-independent ARF/VEGF axis is of major clinical importance since p53 is inactivated in ∼50% of human cancers (Sherr, 2006).

A further aspect that needs to be addressed is the role of ARF in spermatogenesis. Deciphering the poorly defined ability of ARF to cross-talk with components of the HR mechanism during this process could provide new insights on other underrated functions of ARF. Specifically, in the case of male germ cells ARF contributes to their genomic integrity during their maturation, but the exact mechanism(s) is still lacking (Churchman et al., 2011). Even more, as ATM also participates in this process and since they are functionally linked (Velimezi et al., 2013), the exact way their function is coordinated throughout this process remains to be defined.

Finally, taking into consideration the role of ARF not only in the involution of HVS in the developing oculus (Thornton et al., 2005) but also in the involution of the mammary gland (Yi et al., 2004), it is plausible that ARF plays an even wider role than that of a tumor suppressor by acting as a potent “tissue remodeling factor” controlling transient histological structures. Studies toward this direction are essential and could open a new research field related with ARF.

## Conflict of Interest Statement

The authors declare that the research was conducted in the absence of any commercial or financial relationships that could be construed as a potential conflict of interest.

## SUPPLEMENTARY MATERIAL

Figure S1 **ARF demonstrates a p53-independent anti-angiogenic activity in a malignant environment. (A)** Evidence for a p53-independent ATM/ARF/VEGF pathway. Human ARF protein (mouse monoclonal antibody, 1:100 dilution, DCS-240, #ab49166, Abcam) was up-regulated in the presence of the selective ATM inhibitor Ku55933 (Ku, #118500, Calbiochem, MERCK) and as previously shown biologically effective (Velimezi et al., 2013). This was associated with a decrease in VEGF protein (rabbit polyclonal antibody, 1:100 dilution, A-20, #sc-152, SantaCruz) levels as assessed by immunoblot analysis in human cancer cells H1299 and HeLa, both of which are devoid of functional p53. Actin (rabbit polyclonal antibody, 1:1000 dilution, Cell Signaling Technology Inc., #4967) served as a loading control. (ATMi: treatment with the Ku55933 chemical inhibition of ATM activity). **(B)** ARF-dependent decreased microvessel density in a malignant environment. The non-small cell lung cancer (NSCLC) cells H1299, which do not express a functional p53 were xenografted in immunocompromised mice and formed tumors (Velimezi et al., 2013). Immunohistochemical (IHC) analysis was carried out using an anti-CD31 antibody in order to evaluate mean microvessel density (MVD) in sections from tumor tissue. MVD is more than twofold decreased in tumors that were injected with a lentiviral vector expressing short-hairpin (sh) RNA targeting ATM (ctl-shRNA/Lenti-shATM) where ARF is known to be upregulated. On the contrary, the MVD in H1299-shARF xenografts, where ATM has been silenced (shARF/Lenti-shATM), is not decreased. Rather, it is comparable to those estimated in ARF-expressing xenografts without lentivirus-mediated silencing of ATM kinase (ctl-shRNA/Lenti-ctl).

## Abbreviations

ARF: alternative reading frame
ATM: ataxia-telangiectasia mutated
DDR: DNA damage response
DMSO: dimethyl sulfoxide
DSBs: doubled-strand breaks
FSH: follicle-stimulating hormone
HA: hyaloid artery
HIF1α: hypoxia-inducible factor 1α
HR: homologous recombination
HVS: hyaloid vascular system
IRES: internal ribosome entry sequence
LH: luteinizing hormone
MEFs: mouse embryonic fibroblasts
MVD: microvessel density
NPM/B23: nucleophosmin
NSCLC: non-small cell lung cancer
P: postnatal day
PDGFβ: platelet-derived growth factor β
PDGFRβ: platelet-derived growth factor receptor β
PHPV: persistent hyperplastic primary vitreous
pRb: retinoblastoma protein
sh: short-hairpin
VEGF: vascular endothelial growth factor
YFP: yellow fluorescent protein.

## References

[B1] Barlow C. Hirotsune S. Paylor R. Liyanage M. Eckhaus M. Collins F. (1996). Atm-deficient mice: a paradigm of ataxia telangiectasia. *Cell* 86 159–171 10.1016/S0092-8674(00)80086-0 8689683

[B2] Barlow C. Liyanage M. Moens P. B. Tarsounas M. Nagashima K. Brown K. (1998). Atm deficiency results in severe meiotic disruption as early as leptonema of prophase I. *Development* 125 4007–4017 9735362 10.1242/dev.125.20.4007

[B3] Bergers G. Benjamin L. E. (2003). Tumorigenesis and the angiogenic switch. *Nat. Rev. Cancer* 3 401–410 10.1038/nrc1093 12778130

[B4] Busch S. E. Gurley K. E. Moser R. D. Kemp C. J. (2012). ARF suppresses hepatic vascular neoplasia in a carcinogen-exposed murine model. *J. Pathol.* 227 298–305 10.1002/path.4024 22430984 PMC3871210

[B5] Cooke H. J. Saunders P. T. (2002). Mouse models of male infertility. *Nat. Rev. Genet.* 3 790–801 10.1038/nrg911 12360237

[B6] Churchman M. L. Roig I. Jasin M. Keeney S. Sherr C. J. (2011). Expression of arf tumor suppressor in spermatogonia facilitates meiotic progression in male germ cells. *PLoS Genet.* 7:e1002157 10.1371/journal.pgen.1002157 PMC3141002 21811412

[B7] Crinò L. Metro G. (2014). Therapeutic options targeting angiogenesis in nonsmall cell lung cancer. *Eur. Respir. Rev.* 23 79–91 10.1183/09059180.00008913 24591665 PMC9487252

[B8] Damalas A. Velimezi G. Kalaitzakis A. Liontos M. Papavassiliou A. G. Gorgoulis V. (2011). Loss of p14^ARF^ confers resistance to heat shock- and oxidative stress-mediated cell death by upregulating β-catenin. *Int. J. Cancer* 128 1989–1995 10.1002/ijc.25510 20549705

[B9] de Stanchina E. McCurrach M. E. Zindy F. Shieh S. Y. Ferbeyre G. Samuelson A. V. (1998). E1A signaling to p53 involves the p19^ARF^ tumor suppressor. *Genes Dev.* 12 2434–2442 10.1101/gad.12.15.2434 9694807 PMC317046

[B10] Eisen T. Sternberg C. N. Robert C. Mulders P. Pyle L. Zbinden S. (2012). Targeted therapies for renal cell carcinoma: review of adverse event management strategies. *J. Natl. Cancer Inst.* 104 93–113 10.1093/jnci/djr511 22235142

[B11] Elson A. Wang Y. Daugherty C. J. Morton C. C. Zhou F. Campos-Torres J. (1996). Pleiotropic defects in ataxia-telangiectasia protein-deficient mice. *Proc. Natl. Acad. Sci. U.S.A.* 93 13084–13089 10.1073/pnas.93.23.13084 8917548 PMC24050

[B12] Fatyol K. Szalay A. A. (2001). The p14ARF tumor suppressor protein facilitates nucleolar sequestration of hypoxia-inducible factor-1alpha (HIF-1alpha) and inhibits HIF-1-mediated transcription. *J. Biol. Chem.* 276 28421–28429 10.1074/jbc.M102847200 11382768

[B13] Ferrara N. Hillan K. J. Gerber H. P. Novotny W. (2004). Discovery and development of bevacizumab, an anti-VEGF antibody for treating cancer. *Nat. Rev. Drug Discov.* 3 391–400 10.1038/nrd1381 15136787

[B14] Freeman-Anderson N. E. Zheng Y. McCalla-Martin A. C. Treanor L. M. Zhao Y. D. Garfin P. M. (2009). Expression of the Arf tumor suppressor gene is controlled by Tgfbeta2 during development. *Development* 136 2081–2089 10.1242/dev.033548 19465598 PMC2685726

[B15] Golding S. E. Rosenberg E. Valerie N. Hussaini I. Frigerio M. Cockcroft X. F. (2009). Improved ATM kinase inhibitor KU-60019 radiosensitizes glioma cells, compromises insulin, AKT and ERK prosurvival signaling, and inhibits migration and invasion. *Mol. Cancer Ther.* 8 2894–902 10.1158/1535-7163.MCT-09-0519 19808981 PMC2761990

[B16] González-Navarro H. Abu Nabah Y. N. Vinué A. Andrés-Manzano M. J. Collado M. Serrano M. (2010). p19ARF deficiency reduces macrophage and vascular smooth muscle cell apoptosis and aggravates atherosclerosis. *J. Am. Coll. Cardiol.* 55 2258–2268 10.1016/j.jacc.2010.01.026 20381282

[B17] Gromley A. Churchman M. L. Zindy F. Sherr C. J. (2009). Transient expression of the Arf tumor suppressor during male germ cell and eye development in Arf-Cre reporter mice. *Proc. Natl. Acad. Sci. U.S.A.* 106 6285–6290 10.1073/pnas.0902310106 19339492 PMC2664155

[B18] Inagaki A. Schoenmakers S. Baarends W. M. (2010). DNA double strand break repair, chromosome synapsis and transcriptional silencing in meiosis. *Epigenetics* 5 255–266 10.4161/epi.5.4.11518 20364103

[B19] Kamijo T. Bodner S. van de Kamp E. Randle D. H. Sherr C. J. (1999). Tumor spectrum in ARF-deficient mice. *Cancer Res.* 59 2217–2222 10232611

[B20] Kawagishi H. Nakamura H. Maruyama M. Mizutani S. Sugimoto K. Takagi M. (2010). ARF suppresses tumor angiogenesis through translational control of VEGFA mRNA. *Cancer Res.* 70 4749–4758 10.1158/0008-5472.CAN-10-0368 20501856

[B21] Kerr B. A. Byzova T. V. (2012). The dark side of the oxidative force in angiogenesis. *Nat. Med.* 18 1184–1185 10.1038/nm.2881 22869186

[B22] Kotsinas A. Papanagnou P. Galanos P. Schramek D. Townsend P. Penninger J. M. (2014). MKK7 and ARF: new players in the DNA damage response scenery. *Cell Cycle* 13 1227–1236 10.4161/cc.28654 24675893 PMC4049959

[B23] Liontos M. Pateras I. S. Evangelou K. Gorgoulis V. G. (2012). The tumor suppressor gene ARF as a sensor of oxidative stress. *Curr. Mol. Med.* 12 704–715 10.2174/156652412800792633 22292438

[B24] Lowe S. W. Sherr C. J. (2003). Tumor suppression by Ink4a-Arf: progress and puzzles. *Curr. Opin. Genet. Dev.* 13 77–83 10.1016/S0959-437X(02)00013-8 12573439

[B25] McKeller R. N. Fowler J. L. Cunningham J. J. Warner N. Smeyne R. J. Zindy F. (2002). The Arf tumor suppressor gene promotes hyaloid vascular regression during mouse eye development. *Proc. Natl. Acad. Sci. U.S.A.* 99 3848–3853 10.1073/pnas.052484199 11891301 PMC122612

[B26] Palmero I. Pantoja C. Serrano M. (1999). p19ARF links the tumour suppressor p53 to Ras. *Nature* 395 125–126 10.1038/25870 9744268

[B27] Quelle D. E. Zindy F. Ashmun R. A. Sherr C. J. (1995). Alternative reading frames of the INK4a tumor suppressor gene encode two unrelated proteins capable of inducing cell cycle arrest. *Cell* 83 993–100 10.1016/0092-8674(95)90214-7 8521522

[B28] Randall L. M. Monk B. J. (2010). Bevacizumab toxicities and their management in ovarian cancer. *Gynecol. Oncol.* 117 497–504 10.1016/j.ygyno.2010.02.021 20363017 PMC5109972

[B29] Saporita A. J. Maggi L. B. Jr. Apicelli A. J. Weber J. D. (2007). Therapeutic targets in the ARF tumor suppressor pathway. *Curr. Med. Chem.* 14 1815–1827 10.2174/092986707781058869 17627519 PMC2859726

[B30] Sherr C. J. (2006). Divorcing ARF and p53: an unsettled case. *Nat. Rev. Cancer* 6 663–73 10.1038/nrc1954 16915296

[B31] Silva R. L. Thornton J. D. Martin A. C. Rehg J. E. Bertwistle D. Zindy F. (2005). Arf-dependent regulation of Pdgf signaling in perivascular cells in the developing mouse eye. *EMBO J.* 24 2803–2814 10.1038/sj.emboj.7600751 16037818 PMC1182246

[B32] Sugimoto M. Kuo M. L. Roussel M. F. Sherr C. J. (2003). Nucleolar Arf tumor suppressor inhibits ribosomal RNA processing. *Mol. Cell.* 11 415–424 10.1016/S1097-2765(03)00057-1 12620229

[B33] Tago K. Chiocca S. Sherr C. J. (2005). Sumoylation induced by the Arf tumor suppressor: a p53-independent function. *Proc. Natl. Acad. Sci. U.S.A.* 102 7689–7694 10.1073/pnas.0502978102 15897463 PMC1129025

[B34] Takubo K. Ohmura M. Azuma M. Nagamatsu G. Yamada W. Arai F. (2008). Stem cell defects in ATM-deficient undifferentiated spermatogonia through DNA damage-induced cell-cycle arrest. *Cell Stem Cell* 2 170–182 10.1016/j.stem.2007.10.023 18371438

[B35] Thornton J. D. Silva R. L. Martin A. C. Skapek S. X. (2005). The Arf tumor suppressor regulates platelet-derived growth factor receptor beta signaling: a new view through the eyes of *Arf^-/-^* mice. *Cell Cycle* 4 1316–1319 10.4161/cc.4.10.2109 16205116

[B36] Través P. G. Luque A. Hortelano S. (2012). Macrophages, inflammation, and tumor suppressors: ARF, a new player in the game. *Mediators Inflamm.* 2012 568783 10.1155/2012/568783 PMC3538382 23316105

[B37] Tsantoulis P. K. Gorgoulis V. G. (2005). Involvement of E2F transcription factor family in cancer. *Eur. J. Cancer* 41 2403–2414 10.1016/j.ejca.2005.08.005 16213134

[B38] Ulanet D. B. Hanahan D. (2010). Loss of p19^Arf^ facilitates the angiogenic switch and tumor initiation in a multi-stage cancer model via p53-dependent and independent mechanisms. *PLoS ONE* 5:e12454 10.1371/journal.pone.0012454 PMC2929208 20805995

[B39] Velimezi G. Liontos M. Vougas K. Roumeliotis T. Bartkova J. Sideridou M. (2013). Functional interplay between the DNA-damage-response kinase ATM and ARF tumour suppressor protein in human cancer. *Nat. Cell Biol.* 15 967–977 10.1038/ncb2795 23851489

[B40] Weber J. D. Jeffers J. R. Rehg J. E. Randle D. H. Lozano G. Roussel M. F. (2000). p53-independent functions of the p19^ARF^ tumor suppressor. *Genes Dev.* 14 2358–2365 10.1101/gad.827300 10995391 PMC316930

[B41] Xu Y. Ashley T. Brainerd E. E. Bronson R. T. Meyn M. S. Baltimore D. (1996). Targeted disruption of ATM leads to growth retardation, chromosomal fragmentation during meiosis, immune defects, and thymic lymphoma. *Genes Dev.* 10 2411–2422 10.1101/gad.10.19.2411 8843194

[B42] Yi Y. Shepard A. Kittrell F. Mulac-Jericevic B. Medina D. Said T. K. (2004). p19ARF determines the balance between normal cell proliferation rate and apoptosis during mammary gland development. *Mol. Biol. Cell* 15 2302–2311 10.1091/mbc.E03-11-0785 15105443 PMC404024

[B43] Zindy F. Eischen C. M. Randle D. H. Kamijo T. Cleveland J. L. Sherr C. J. (1998). Myc signaling via the ARF tumor suppressor regulates p53-dependent apoptosis and immortalization. *Genes Dev.* 12 2424–2433 10.1101/gad.12.15.2424 9694806 PMC317045

