# Supplementary figures and images for: ARF: a versatile DNA damage response ally at the crossroads of development and tumorigenesis
Source: Front Genet. 2014 Jul 22;5:236. doi: 10.3389/fgene.2014.00236 (PMC4106421; doi:10.3389/fgene.2014.00236)

Suppl Figure 1

a.

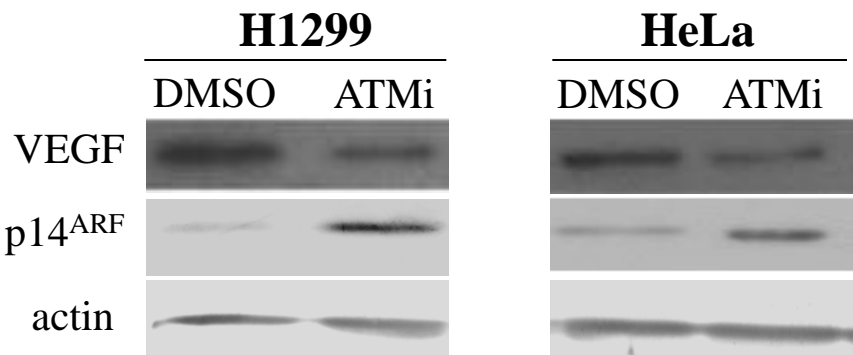

b.

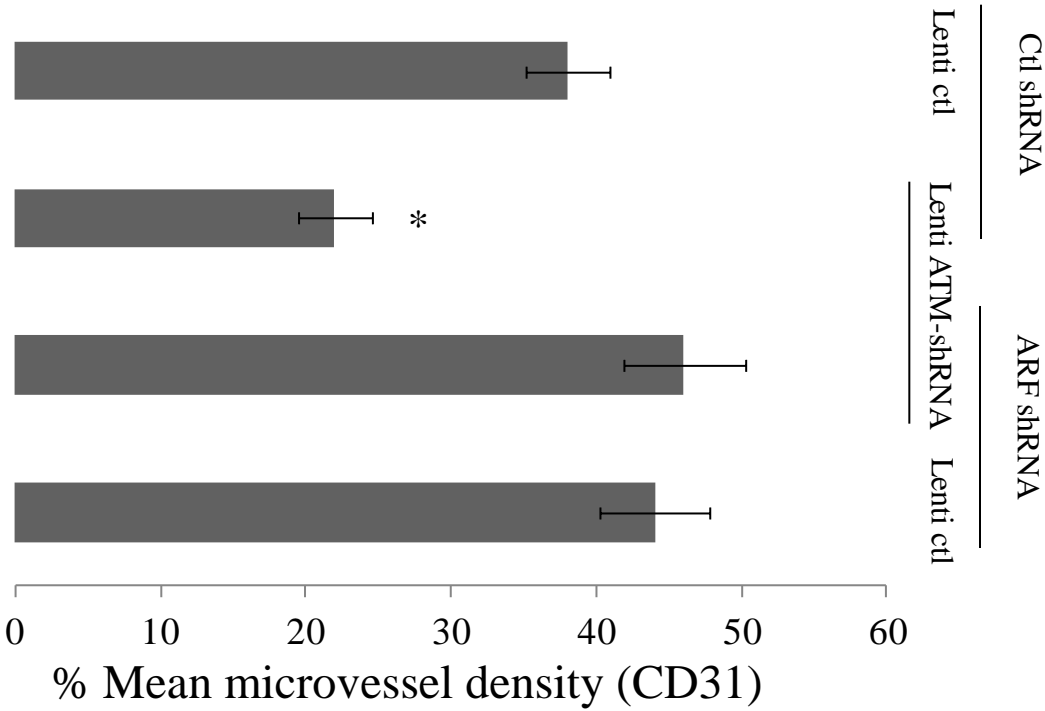

Supplement: Figure S1 — ARF demonstrates a p53-independent anti-angiogenic activity in a malignant environment. (A) Evidence for a p53-independent ATM/ARF/VEGF pathway. Human ARF protein (mouse monoclonal antibody, 1:100 dilution, DCS-240, #ab49166, Abcam) was up-regulated in the presence of the selective ATM inhibitor Ku55933 (Ku, #118500, Calbiochem, MERCK) and as previously shown biologically effective (Velimezi et al., 2013). This was associated with a decrease in VEGF protein (rabbit polyclonal antibody, 1:100 dilution, A-20, #sc-152, SantaCruz) levels as assessed by immunoblot analysis in human cancer cells H1299 and HeLa, both of which are devoid of functional p53. Actin (rabbit polyclonal antibody, 1:1000 dilution, Cell Signaling Technology Inc., #4967) served as a loading control. (ATMi: treatment with the Ku55933 chemical inhibition of ATM activity). (B) ARF-dependent decreased microvessel density in a malignant environment. The non-small cell lung cancer (NSCLC) cells H1299, which do not express a functional p53 were xenografted in immunocompromised mice and formed tumors (Velimezi et al., 2013). Immunohistochemical (IHC) analysis was carried out using an anti-CD31 antibody in order to evaluate mean microvessel density (MVD) in sections from tumor tissue. MVD is more than twofold decreased in tumors that were injected with a lentiviral vector expressing short-hairpin (sh) RNA targeting ATM (ctl-shRNA/Lenti-shATM) where ARF is known to be upregulated. On the contrary, the MVD in H1299-shARF xenografts, where ATM has been silenced (shARF/Lenti-shATM), is not decreased. Rather, it is comparable to those estimated in ARF-expressing xenografts without lentivirus-mediated silencing of ATM kinase (ctl-shRNA/Lenti-ctl). [file Presentation_1.PDF]
